# PILLAR: ParaspInaL muscLe segmentAtion pRoject - a comprehensive online resource to guide manual segmentation of paraspinal muscles from magnetic resonance imaging

**DOI:** 10.1186/s12891-023-07029-x

**Published:** 2023-11-23

**Authors:** Meagan Anstruther, Bianca Rossini, Tongwei Zhang, Terrance Liang, Yiming Xiao, Maryse Fortin

**Affiliations:** 1 Department Health Kinesiology and Applied Physiology, Concordia University, 7141 Sherbrooke Street W, SP-165.29, Montreal, QC H4B 1R6 Canada; 2 Department of Computer Science and Software Engineering, Concordia University, Montreal, QC Canada; 3 School of Health, Concordia University, Montreal, QC Canada; 4 Centre de Recherche Interdisciplinaire en Réadaptation (CRIR), Montreal, QC Canada

**Keywords:** Lumbar spine, Magnetic resonance imaging, Fat infiltration, Manual segmentation, Low back pain

## Abstract

**Background:**

There is an increasing interest in assessing paraspinal morphology and composition in relation to low back pain (LBP). However, variations in methods and segmentation protocols contribute to the inconsistent findings in the literature. We present an on-line resource, the **P**arasp**I**na**L** musc**L**e segment**A**tion p**R**oject (PILLAR, https://projectpillar.github.io/), to provide a detailed description and visual guide of a segmentation protocol by using the publicly available ITK-SNAP software and discuss related challenges when performing paraspinal lumbar muscles segmentations from magnetic resonance imaging (MRI).

**Methods:**

T2-weighted and corresponding fat-water IDEAL axial MRI from 3 males and 3 females (2 chronic LBP and 1 control for each sex) were used to demonstrate our segmentation protocol for each lumbar paraspinal muscle (erector spinae, lumbar multifidus, quadratus lumborum and psoas) and lumbar spinal level (L1-L5).

**Results:**

Proper segmentation requires an understanding of the anatomy of paraspinal lumbar muscles and the variations in paraspinal muscle morphology and composition due to age, sex, and the presence of LBP or related spinal pathologies. Other challenges in segmentation includes the presence and variations of intramuscular and epimuscular fat, and side-to-side asymmetry.

**Conclusion:**

The growing interest to assess the lumbar musculature and its role in the development and recurrence of LBP prompted the need for comprehensive and easy-to-follow resources, such as the PILLAR project to reduce inconsistencies in segmentation protocols. Standardizing manual muscle measurements from MRI will facilitate comparisons between studies while the field is progressively moving towards the automatization of paraspinal muscle measurements for large cohort studies.

## Introduction

Low back pain (LBP) is a very common symptom and now the leading cause of disability worldwide [1]. While the precise mechanisms underlying LBP remain largely unknown, there is a global consensus as to its multifactorial aetiology including, but not limited to, biophysical factors, psychological factors and societal factors [2, 3]. Despite recognition of these factors, there remains limited options for effective conservative management programs [4, 5].

Increased paraspinal muscle fatty infiltration has been associated with the presence and severity of spinal pain and dysfunction [6–9]. This has lead to a growing interest in using quantitative imaging measures of muscle composition to improve phenotyping and prognosis and to provide a necessary biomarker towards informing and measuring therapeutic success. However, findings from recent reviews reporting on the association between image-based measures of paraspinal muscle morphology and composition with LBP and related spinal pathologies remain conflicting and inconclusive [10–12]. Important variations including inconsistent segmentation protocols for the lumbar musculature (e.g., undefined borders, inclusion or exclusion of epimuscular fat, and use of proprietary imaging software) and image perceptions by the raters likely contributed to the inconsistencies in the literature. Hodges et al. [13] recently published a review and consensus-based recommendations to address these inconsistencies and work towards the standardisation of imaging-based measures of paraspinal muscles. Future studies should implore to follow these recommendations to allow for easier comparisons between studies.

When it comes to which image navigation software to use to perform paraspinal muscle segmentation, several factors can arise, including accessibility, costs, ease-of-use, and the goals of research. ITK-SNAP (www.itksnap.org) is a user-friendly, free open-source medical image segmentation software that has been used in several domains including cardiac, dental, brain, and spinal applications for imaging analysis and to aid in diagnosis and surgery. While relatively new to the LBP community, this software allows for segmentations in simultaneous consideration of multiple co-aligned image contrast, which is useful when examining fat vs. water derived magnetic resonance images (MRI) of muscles. ITK-SNAP also provides easy labeling tools allowing for clear identification of various muscles and structures in a single image, in addition to 3D segmentation visualization and volumetric measurement.

The **P**arasp**I**na**L** musc**L**e segment**A**tion p**R**oject (PILLAR) is a comprehensive on-line resource of protocols and tutorials designed to provide new researchers with the information and tools to perform educated manual segmentations of lumbar musculature in ITK-SNAP. Through PILLAR, our segmentation protocol, anatomy, and borders for lumbar multifidus (LM), erector spinae (ES), quadratus lumborum (QL), and psoas (PS) are clearly defined with both detailed description and visualization. In addition, videos for the segmentation of each muscle and step-by-step guide on how to use ITK-SNAP are provided, the inclusion versus exclusion of epimuscular fat in the region of interest (ROI) is discussed, and guidelines for reporting measurement information in papers are also supplied. Furthermore, this project shows side-by-side comparisons of healthy, pathological, and aged pathological conditions (e.g., chronic LBP) in both males and females across all lumbar levels.

## Objectives

The primary objective of this project is to provide a detailed description of our segmentation protocol, introduce the uses of ITK-SNAP to the LBP research community for paraspinal muscle segmentation, and provide a clear visual guide to help train new raters and facilitate larger scale comparison between studies. A secondary objective is to discuss key challenges when segmenting paraspinal muscle from axial MR images and highlight differences in paraspinal muscle morphology and composition related to age, sex and spinal pathology.

## Methods

### Image acquisition and reconstruction

MRI images of 3 females (27-year-old control, 32-year-old with chronic LBP, and 51-year-old with chronic LBP) and 3 males (29-year-old control, 34-year-old with chronic LBP, and 60-year-old with chronic LBP) were selected from previous ongoing research projects. Ethics approval was obtained from the Central Ethics Research Committee of the Quebec Minister of Health and Social Services and all subjects provided informed consent. All methods were carried in accordance with relevant guidelines and regulations.

All subjects underwent a routine lumbosacral MRI evaluation using a 3T GE magnet (Milwaukee, WI, USA) and started phased array body coil. Axial T2-weighted and multi-echo IDEAL (Lava-flex, 2 echo sequence) were acquired from L1 to L5 using the following MR parameters; 4-mm slice thickness, 180-mm^2^ field of view and 512 × 512 matrix. Slices at the mid-disc from L1-L5 were selected from T2-weighted and IDEAL fat-water images. If needed, multiplanar reconstruction (3D MPR) using the HOROS software (Version 4.0.0) was used at the L4 and L5 levels to position the image slices perpendicular to the long axis of the paraspinal musculature. ITK-SNAP (Version 3.8.0) was then used to segment LM, ES, QL, and psoas. T2-weighted images were segmented separately from fat/water images. Some images were darker than others and their contrast needed to be adjusted to visualize the borders properly. Finding the borders on the fat and water images may be more difficult especially if there is little fat in the image. Muscle is dark and fat is bright in fat images, whereas muscle is a grainy gray and fat is darker in water images.

### Anatomical landmarks for segmentation

The following landmarks were used for segmentation purposes. The LM muscle has borders along the spinous process, lamina, intermuscular fascial border with the erector spinae, and the LM epimysium that is distinct from the thoracolumbar fascia (TLF) and adipose tissue. The ES shares borders with LM, the tip of the zygoapophyseal joint, intermuscular fascial border between QL, and along the aponeurosis that is distinct from the TLF and subcutaneous adipose tissue. When present, epimuscular fat was included in the ES ROI. QL extends along the fascial borders with ES and psoas, 12th rib at L1, perirenal fascia from L2-L4, and iliac crest at L4. Psoas runs along the intervertebral disc, interfascial border with the viscera, kidneys, and the intermuscular fascia between QL and ES depending on the shape of the muscle. Additional information and anatomical landmarks used to identify the medial, anterior, lateral and posterior border of each muscle [14, 15], as well as further segmentation tips are presented in Table 1.

**Table 1:** Anatomical landmarks for each lumbar paraspinal muscle and segmentation tips

Paraspinal Muscle	Anatomical landmarks for segmentation
Lumbar multifidus (LM)	• **Medial border** - most superficial aspect of the spinous process as it leads into the lamina including any fat located along the spinous process • **Anterior border** - follow the lamina towards the zygapophyseal joint • **Lateral border** - intermuscular fascial line between LM and ES from the mammillary process to the small visible indentation in the subcutaneous tissue along the posterior aspect • **Posterior border** - along the LM epimysium that is distinct from the thoracolumbar fascia (TLF) and adjacent adipose tissue *Additional tips for segmentation*: Intermuscular fat next to the spinous process is included as part of the LM region of interest (ROI). If fat is present along the lateral border (i.e. between LM and ES), this fat **is not** included as part of the LM ROI (i.e. this fat will included as part of the ES ROI). If only a small amount of fat is present along the lateral border and distinction between LM and ES is unclear, look for small pockets of fat there the anterial and lateral borders meet or where the posterior and lateral borders meet to see the beginning of a thin white line - the trajectory can be followed through the dark patches to assume the lateral border. Ensure the lateral border is the fascial border between LM and ES and not the border between the longissimus and iliocostalis muscles that make up the ES.
Erector Spinae (ES)	• **Medial border** - intermuscular fascial line between LM and ES to the zygapophyseal joint or mammillary process • **Anterior border** - along the transverse process and intermusuclar fascial border between ES and QL • **Lateral border** - rounded edge of the fascial border of the iliocostalis muscle • **Posterior border** - along the ES muscle and aponeurosis which is distinct from the TLF and adjacent subcutaneous adipose tissue *Additional tips for segmentation*: If fat has accumulated along the medial border (i.e. between LM and ES), this fat is included as part of the ES ROI. The anterior border leading towards the lateral border runs along the QL. Sometimes there is a clear fascial line to separate the ES from the QL, but often there is not. If the border is not clear, follow the rounded edge at the lateral border and work towards the anterior border at the tip of the zygapophyseal joint. This point of ES may also come into contact with the posterior border of the psoas muscle in the upper lumbar levels. There is still debate whether to *include* or *exclude* epimuscular fat when present (i.e., the white fat-filled “tents” between longissimus and iliocostalis or it may span the length of the posterior border) for the posterior border of ES. With our segmentation protocol, epimuscular fat, was *included* as part of the ES ROI (refer to Fig. 3A).
Quadratus Lumborum (QL)	• **Medial border** - may extend all the way towards the mammillary process at the tip of the ES border or may come to a point meeting psoas and ES depending on the lumbar level investigated • **Anterior border** - intermuscular fascial line between QL and psoas • **Lateral border** − 12th rib at the level of L1, posterior perirenal fascia at the level of L2-L4, iliac crest at the level of L4 • **Posterior border** - intermuscular fascial line between QL and ES *Additional tips for segmentation*: The QL is generally an elongated muscle with the posterior border being straight. If the border is not clear, follow the line of the small fat accumulation where the lateral and posterior borders meet with ES. At L5, QL is no longer visible on MR images due to its insertion point being the superior aspect of the iliac crest.
Psoas (PS)	• **Medial border** - the intervertebral disc • **Anterior border** - interfascial border with the viscera and may border several blood vessels (e.g. splenic artery, inferior vena cava, etc.) depending on the level and whether on the right or left side • **Lateral border** - approximately L1-L4 borders the kidneys, L5 borders the posterior perirenal fascia • **Posterior border** - intermuscular fascial line between psoas and either QL or ES depending on the shape of the muscles in the image and the lumbar level in question *Additional tips for segmentation*: The PS is generally round with a possible elongation at the posterior border at the lower lumbar levels. At the upper lumbar levels, PS may be narrower as the muscle begins at L1-L3.

**Fig. 1:**
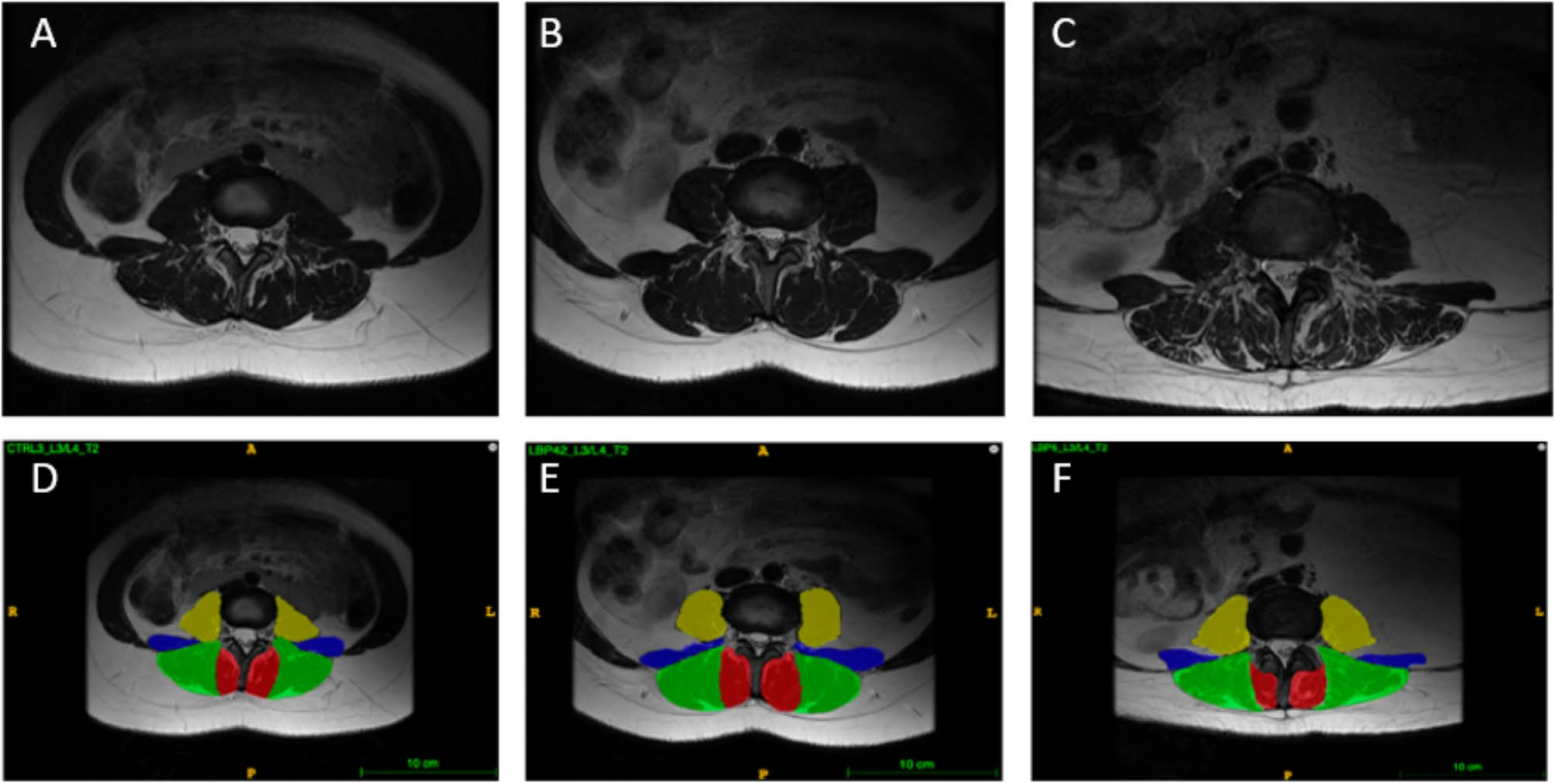
All images are T2-weighted images at the L3/L4 level in females. The first column (A & D) are images of the control. The middle column (B & E) are images of an age-matched individual with LBP. The third column (C & F) are images of the older individual with LBP. D, E, and F include the segmentations of the lumbar multifidus (red), erector spinae (green), quadratus lumborum (blue), and psoas (yellow) muscles

**Fig. 2:**
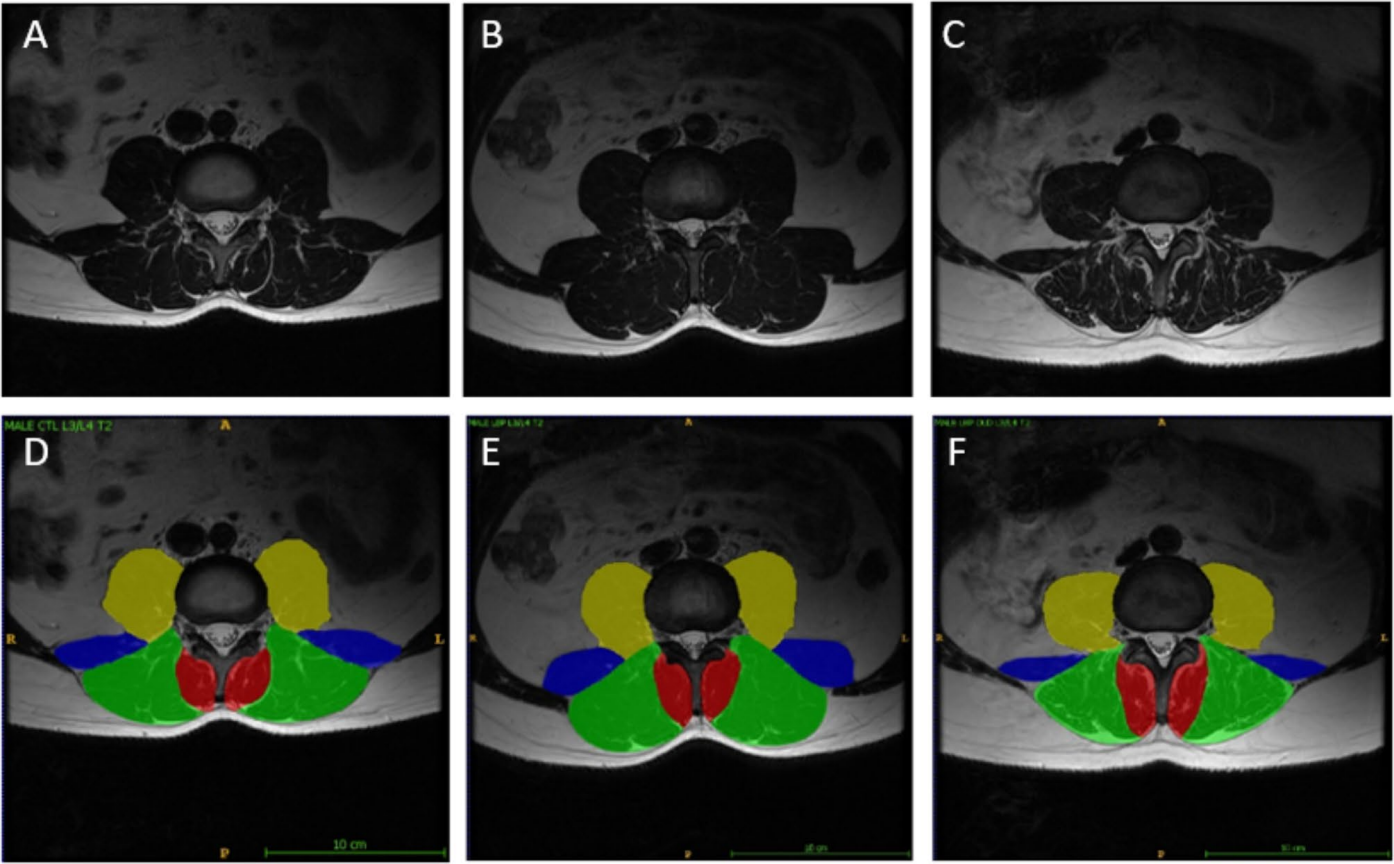
All images are T2-weighted images at the L3/L4 level in males. The first column (A & D) are images of the control. The middle column (B & E) are images of an age-matched individual with LBP. The third column (C & F) are images of the older individual with LBP. D, E, and F include the segmentations of the lumbar multifidus (red), erector spinae (green), quadratus lumborum (blue), and psoas (yellow) muscles

**Fig. 3:**
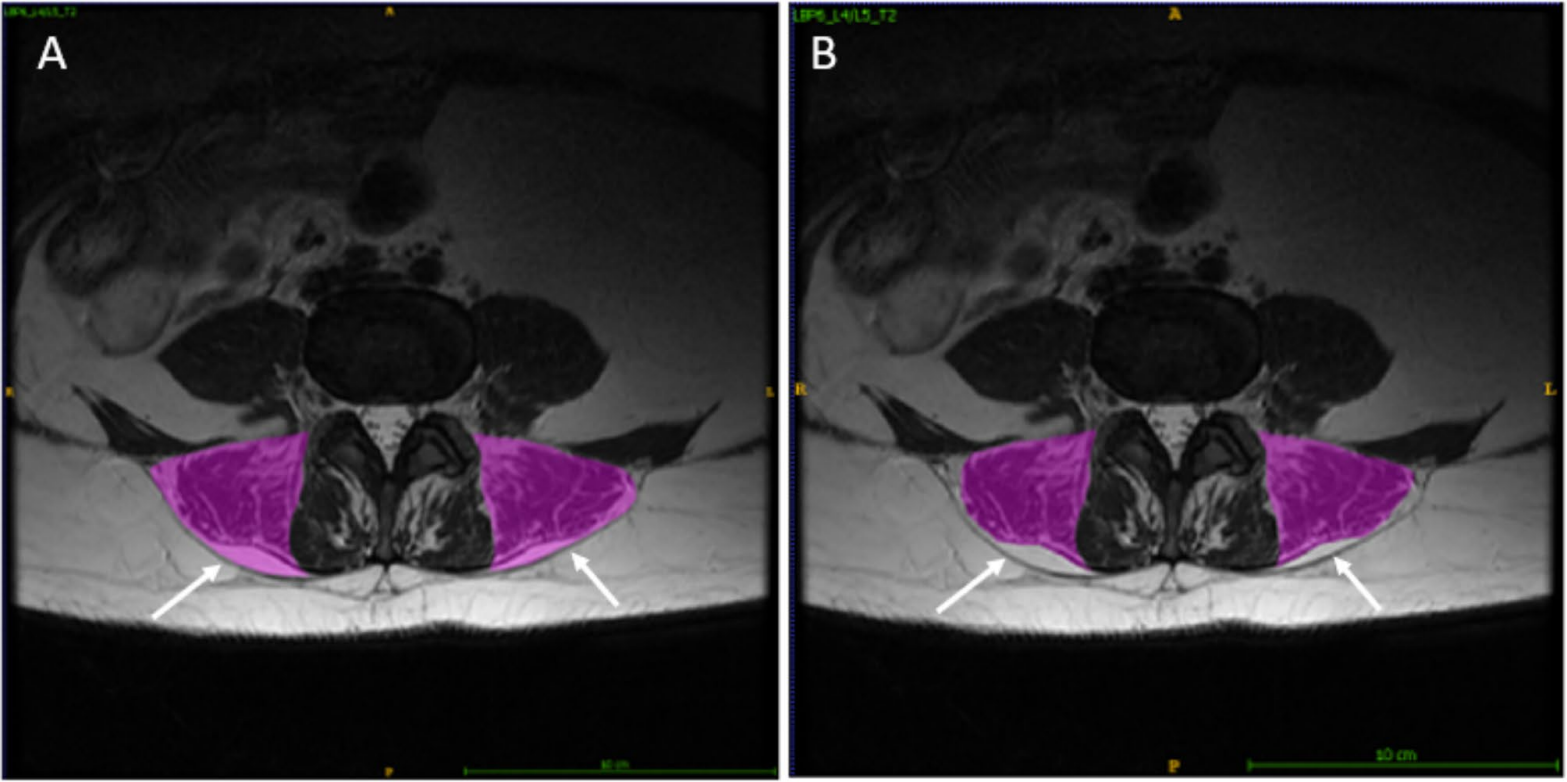
T2-weighted images at the L4/L5 level in a female with LBP. (A) includes the epimuscular fat in the CSA measurement. (B) excludes the epimuscular fat in the CSA measurement

To provide researchers both new and familiar with muscular segmentation of the lumbar spine, a tutorial website for the PILLAR project was created to accompany this paper (https://projectpillar.github.io/). The website provides detailed anatomy of the lumbar musculature as well as the suggested borders for segmentation. Furthermore, several video tutorials on how to use the ITK-SNAP software, example images of manual segmentations on 3 females (Figs. 1) and 3 males (Fig. 2) and differences between the inclusion and exclusion of epimuscular fat (Fig. 3), and the standardized recommendations for manual segmentation can be found on the website. Each section of this online tutorial can be easily navigated using the menu bar on the left-hand side. More segmentations may be added in the future to show different conditions researchers may come across and will be updated accordingly as new research is published.

## Discussion

### Challenges with segmentation

Several challenges arise when investigating different populations and pathologies. Individuals who are older are more likely to have increased intramuscular and epimuscular fat [14]. In addition, females are also more likely to have increased epimuscular and intramuscular fat compared to males, which is likely due to females generally having a higher body fat % [16]. Lastly, those with LBP and disc degeneration typically present with increased intramuscular fat, which is believed to be related to decreased function and pain [6, 17]. The presence of intramuscular fat on MRI images may make determining muscular borders easier since a clear white line may appear on the images where the fat builds up along fascial borders. If there are large amounts of intramuscular fat, it can be difficult to determine where one muscle begins and another ends because there are multiple white lines. Epimuscular fat typically presents itself along the ES between the longissimus and iliocostalis muscles and is commonly referred to as a fat “tent”. To date, studies have both included and excluded epimuscular fat in their paraspinal muscle measurements. Researchers must clearly define in their methods whether epimuscular fat was included or excluded in the ROI as it will affect both size and muscle composition measurements [18]. In this project, our segmentation protocol included epimuscular fat if present. However, images demonstrating the difference between the inclusion and exclusion of epimusuclar fat can be found on the PILLAR Project website. Knowing the anatomy of the muscles, the shape they typically take on at certain lumbar levels, and understanding the widely accepted borders for these muscles will aid in deciding the path to follow to segment the muscle.

The paraspinal musculatures also differ between younger and older populations, sex, and individuals with the presence of LBP or other spinal pathologies. Older individuals, females, and those with LBP tend to present with increased intramuscular fat. It can be difficult to find the appropriate landmarks on MRIs during segmentation when there is little fat, especially at the borders between muscles (e.g., LM and ES, ES and QL). Males tend to have larger and thicker LM compared to females in both general and athletic populations. Individuals with LBP have presented with decreased LM cross-sectional area and thickness compared to controls.

The presence of LBP and lumbar pathologies has been associated with paraspinal muscle atrophy (e.g., reduced size) and side-to-side asymmetry [18–21]. In some cases, this can also make paraspinal muscle segmentation more difficult. Varying sizes between the right and left sides, especially with LM and QL, can lead to improper segmentation in an unconscious attempt to make both sides equal. It is important in these situations to be familiar with the muscular borders and to follow those borders for the muscle being segmented. While it is important to compare left and right sides to help with the general shape, they may not always be identical. Of note, differences in MF and ES shape between males and females have also been reported [21, 22].

### Future directions

With the increased interest and greater number of researchers investigating the lumbar musculature and its role in the presence of LBP and related spinal pathologies using imaging, there are bound to be inconsistencies between measures, software, and protocols that may hinder proper comparisons between studies and populations. Hodges et al. [13] provided a clear set of recommendations with regards to physiological/pathological, confounding factors, and measurements issues that should be taken into consideration when planning an imaging study investigating lumbar musculature. The PILLAR project is adding to the current literature by providing a clear visual guide, tutorials and resourceful tool to facilitate paraspinal muscle segmentation. Not only will consistency in segmentation protocols aid in comparing studies and clinical populations, but researchers utilizing the same software to perform segmentations will also allow for easier comparisons between studies.

Segmentation is moving towards automated segmentation in many fields of research, which is segmentation completed wholly by a program. Manual segmentation is completed wholly by the researcher. In order to develop an automated segmentation program, large databases must be acquired through manual segmentation [23, 24]. As the program develops and learns from these manually segmented images, it is necessary to verify and edit boundaries, which is what is known as semi-automated segmentation (partial completion by a program, partial completion by a researcher). The PILLAR project provides the tools to aid in the creation of a large database of paraspinal muscle segmentations to move towards automated segmentation. Compared to manual segmentation, automated segmentation is faster, easier, and may allow for easier access to results, especially if it can be integrated to clinical settings.

Furthermore, segmentation is the first step towards grading any disease. While the goal of this paper was not identifying key elements to distinguishing mild, moderate, and severe LBP, utilizing the same segmentation software and protocols discussed here can provide researchers a consistent means of identifying the level of severity of LBP or other pathologies based on paraspinal musculature characteristics (i.e. intramuscular fat and muscle size). This may aid in predicting or identifying individuals at risk of experiencing mild, moderate, or severe LBP or other lumbar spine pathologies associated with paraspinal musculature characteristics.

There are several imaging software commonly used in lumbar musculature segmentation, such as 3D Slicer, ImageJ, and Horos. ITK-SNAP is a free program commonly used in brain, cardiac, and spine segmentation, and while segmentation can be done in any of the mentioned programs, ITK-SNAP provides the ability to examine musculature in a 3D view and calculate volumetric measurements, which has been rarely done to date when investigating the lumbar musculature.

## Data Availability

The data and resources generated from this work is available on the following website: https://projectpillar.github.io/.

## List of abbreviations

LBP: low back pain
PILLAR: **P**arasp**I**na**L** musc**L**e segment**A**tion p**R**oject
MRI: Magnetic Resonance Imagin
LM: Lumbar multifidus
ES: Erector Spinae
QL: Quadratus lumborum
PS: Psoas
ROI: Region of interest
MPR: multiplanar reconstruction
TLF: Thoracolumbar fascia
